# The Symptom-Checklist-K-9 (SCL-K-9) Discriminates between Overweight/Obese Patients with and without Significant Binge Eating Pathology: Psychometric Properties of an Italian Version

**DOI:** 10.3390/nu12030674

**Published:** 2020-03-01

**Authors:** Claudio Imperatori, Emanuela Bianciardi, Cinzia Niolu, Mariantonietta Fabbricatore, Paolo Gentileschi, Giorgio Di Lorenzo, Alberto Siracusano, Marco Innamorati

**Affiliations:** 1 Cognitive and Clinical Psychology Laboratory, Department of Human Science, European University of Rome, Via degli Aldobrandeschi 190, 00163 Roma, Italy; mfabbricatore@tiscali.it (M.F.); Marco.Innamorati@unier.it (M.I.); 2 Psychiatric Chair, Department of Systems Medicine, University of Rome “Tor Vergata”, Via Cracovia, 50, 00133 Roma, Italy; bianciardi@med.uniroma2.it (E.B.); niolu@med.uniroma2.it (C.N.); di.lorenzo@med.uniroma2.it (G.D.L.); siracusano@med.uniroma2.it (A.S.); 3 Bariatric Surgery Unit, Department of Experimental Medicine and Surgery, University of Rome “Tor Vergata”, Via Cracovia, 50, 00133 Roma, Italy; gentileschi.paolo@gmail.com; 4 IRCCS Fondazione Santa Lucia, Via Ardeatina 306, 00179 Roma, Italy

**Keywords:** binge eating, SCL-K-9, obesity, overweight, bariatric surgery candidates, obesity, psychopathology

## Abstract

A general personality and psychopathology evaluation is considered to be crucial part of the multidisciplinary assessment for weight-related problems. The Symptom Checklist-90-Revised (SCL-90-R) is commonly used to assess general psychopathology in both overweight and obese patients seeking weight-loss treatment. The main purpose of the present research was to investigate the psychometric properties of the brief form of the SCL-90-R (i.e., the SCL-K-9) in a clinical sample (*N* = 397) of patients seeking weight-loss treatment (i.e., bariatric surgery and a nutritional weight-loss program). The results of the confirmatory factor analysis supported a one-factor solution of the SCL-K-9, with all nine items loading significantly on the common latent factor (lambdas ≥ 0.587). The ordinal α (= 0.91), the inter-item mean indices of correlation (*r_ii_* = 0.53), and the convergent validity were also satisfactory. A receiver operating characteristic curves procedure showed that both SCL-90-R and SCL-K-9 were able to classify patients with and without significant binge eating pathology according to the Binge Eating Scale (BES) total score. Overall, our results suggest that the SCL-K-9 has adequate psychometric properties and can be applied as a short screening tool to assess general psychopathology in overweight/obese individuals seeking weight-loss treatment and at follow-up interviews when time restraints preclude the use of the full-length form.

## 1. Introduction

Obesity (i.e., body mass index (BMI) ≥ 30 kg/m^2^) and overweight (BMI ≥ 25 kg/m^2^) are serious prevalent health concerns [1] currently affecting over one-third of the world’s population [2]. Indeed, obesity, especially severe obesity (i.e., BMI ≥ 40), is strongly related to morbidity and mortality risk [3,4]. Moreover, weight-loss treatment-seeking individuals are often characterized by dysfunctional eating patterns, especially binge eating [5,6,7,8], as well as high rates of psychopathology, particularly mood and anxiety disorders and body image concerns [6,9,10]. Therefore, a general personality and psychopathology evaluation is considered to be a crucial part of the multidisciplinary assessment for weight-related problems [11,12]. Although the association between psychopathology and the success of weight-loss treatments is controversial [6,13,14], it has often been reported [15,16,17,18,19,20,21,22] that mental health issues (e.g., binge eating disorder) are associated with several aspects of treatment outcomes (e.g., less weight loss, weight regain, and higher drop-out rates). In particular, binge eating disorder in bariatric surgery candidates may predict the development of problematic eating behaviors after surgery [23]. Therefore, brief screening tools to evaluate psychopathology could be extremely useful for clinicians as a first step of a more in-depth assessment procedure.

The Symptom Checklist-90-Revised (SCL-90-R) [24] is one of the most commonly used self-rating measures for assessing general psychopathology in patients seeking weight-loss treatment, such as bariatric surgery or a nutritional weight-loss program [11,12,25,26]. It comprises 90 items rated on a 5-point Likert scale (from “0” to “4”) investigating the severity of nine main psychopathological dimensions over the previous week, including somatic symptoms, interpersonal sensitivity, obsessive–compulsive behaviors, anxiety and depressive symptoms, hostility, phobic symptoms, paranoid tendencies, and psychoticism. Furthermore, this scale proposes a global severity index (GSI-90) as an index of overall psychological distress, with higher scores reflecting higher levels of psychopathological distress as well as a greater severity of self-reported symptoms [27]. 

Several short forms of the SCL-90-R have been proposed and validated since the 1980s in order to provide a more economical instrument [28]. Among these versions, the Symptom-Checklist-K-9 (SCL-K-9), composed of the nine items of the SCL-90-R (#24, #28, #31, #34, #43, #57, #58, #75, #77) that exhibit the highest item-total correlation, was proposed as an efficient and unidimensional screening tool representing all of the original symptom subscales of the SCL-90-R [29]. It was initially validated by a representative sample of German individuals (*N* = 2,057), where satisfactory internal reliability (Cronbach α = 0.87), as well as acceptable convergent validity with other measures of psychopathology (e.g., depression and anxiety), were demonstrated [29].

Satisfactory psychometric properties, including a one-factor structure, adequate internal consistency, and convergent validity with psychopathology were also detected in Ukrainian [30] and German [31] general populations. For example, Petrowski et al. [31] recently documented the fit of a one-factor model (Confirmatory Fit Index (CFI) = 0.949; Tucker Lewis Index (TLI) = 0.932; Root Mean Square Error of Approximation (RMSEA) = 0.053). Results of invariance across gender and age groups, satisfactory internal consistency, and strong associations with both somatic (*r* = 0.60) and depressive symptoms (*r* = 0.71) were also reported [31].

These findings suggest that the SCL-K-9 is an adequate and valid assessment tool that can be used when time restraints preclude the use of the full-length form, such as in large epidemiological cohort surveys and at follow-up interviews [28,29,30,31,32]. However, as observed by Petrowski and coworkers [31], the investigation of the psychometric properties of SCL-K-9 in clinical samples is still an underdeveloped research area. Furthermore, to the best of our knowledge, no previous studies have investigated the psychometric properties and clinical utility of this measure in overweight/obese treatment-seeking sample populations. The use of validated psychological questionnaires to evaluate general psychopathology is recommend as part of the wider multidisciplinary assessment for weight-related problems [11,12]. 

Thus, the purposes of the current research were to propose an Italian adaptation of the SCL-K-9 and to explore its dimensionality and psychometric properties in a clinical sample of obese and overweight patients seeking weight-loss treatment (i.e., bariatric surgery and nutritional weight-loss programs). Another aim was to investigate whether, by using a receiver operating characteristic (ROC) curve procedure, the SCL-K-9 could be able to categorize patients with and without significant binge eating pathology according to the Binge Eating Scale (BES) total score [33] compared to the original SCL-90-R.

## 2. Material and Methods 

### 2.1. Participants 

The present data were extracted from a database used in a previous study by our research team [34]. Briefly, the study participants were 397 overweight and obese patients (291 women and 106 men) who were referred to a medical center in Rome (Italy) specializing in nutritional weight-loss treatment (*N* = 122) or to the center for Bariatric Surgery Unit at the University of Rome “Tor Vergata” (*N* = 275). All study participants were examined at the time of study entry; inclusion/exclusion criteria are described elsewhere [34]. This research was approved by the ethics committee of the University of Rome “Tor Vergata”, in line with the Helsinki declaration standards.

### 2.2. Self-report Measures

After enrolment, the Italian version of the Binge Eating Scale (BES) [8] and the SCL-90-R [35] were administered to all patients. Sociodemographic and clinical data were detected from medical records.

The BES [33] is a 16-item self-report questionnaire widely used to assess binge eating pathology (i.e., behavioral and cognitive/emotional symptoms). The total score ranges from 0 to 46 (with higher scores reflecting more severe binge eating pathology), and three different levels of severity were proposed [36,37]: (1) No significant binge eating (i.e., BES total score ≤ 17), (2) moderate binge eating (i.e., total score ranging from 18 to 26), and (3) severe binge eating (i.e., BES total score ≥ 27). Satisfactory psychometric properties (e.g., good internal consistency and factorial structure) have been widely reported in both surgical and nonsurgical patients [8,38,39,40]. The internal consistency in the present study was α = 0.90.

The SCL-90-R [24] is a 90-item self-report measure used to assess general psychopathology and overall psychological distress. In the present sample, the internal consistency was α = 0.94 for the GSI-90. According to previous studies [29,30], the SCL-9-K was obtained on the basis of SCL-90-R items. The GSI-K-9 is the total score of the SCL-9-K. 

### 2.3. Statistical Analyses

The SPSS 18.0 statistical package for the social sciences (IBM, Chicago, USA) was used to perform all of the analyses. Compared to our previous study [34], 6 participants were excluded due to missing items on the SCL-K-9. 

In order to assess the psychometric properties of the SCL-K-9 at the item level, we reported corrected item-total correlations and descriptive statistics (means, standard deviations, skewness, and kurtosis). As a measure of reliability, we reported the ordinal *α* [41] and the inter-item mean correlation (*r_ii_*). The convergent validity values of the BES, GSI-90, and SCL-90-R subscales were assessed using Pearson’s *r* correlation coefficients. Furthermore, associations with age and BMI were investigated. Sex differences were also evaluated using a *t*-test for independent samples. 

Data were submitted to a confirmatory factor analysis (CFA) using Mplus 6.0 (Los Angeles, CA, USA) [42] and a Weighted Least Square Mean and Variance Adjusted (WLSMV) estimator with a polychoric correlation matrix after calculating Mardia’s multivariate asymmetry skewness and kurtosis statistics [43]. The model fit was assessed using the following indices: (1) the RMSEA, as index of absolute fit, with values between 0.05 and 0.08 suggesting good adequacy of the model and values below 0.05 demonstrating strong evidence of absolute fit [44]; (2) the Weighted Root Mean Square Residual (WRMR), where values less than 1.0 indicated good model fit [45,46]; (3) the chi-squared (*χ^2^*) test, where p-values greater than 0.05 indicated that the model was adequate for the data; and (4) TLI as a measure of a relative model-fit compared to the “null” model, where values > 0.950 were recommended for a good model fit [46].

Lastly, in order to assess the performance of the SCL-K-9 in classifying patients according to their current binge eating severity, we performed a ROC test procedure [47]. A ROC curve is a two dimensional depiction of test performance [48], with the main outcome variable being the area under the ROC curve (AUC), which reflects the probability that a randomly sampled respondent would be correctly categorized [49]. The AUC directly represents the overall accuracy of the instrument in categorizing a sample where values of ≥ 0.70 are considered to be satisfactory [50]. The Youden Index [51] was calculated in order to detect the maximum thresholds of both sensitivity (i.e., the proportion of subjects who have the target condition and exhibit positive test results) and specificity (i.e., the proportion of subjects without the target condition and exhibit negative test results). 

## 3. Results

The patients had an average age of 43.40 years (SD = 12.02; range: 18–73) and the mean BMI was 40.32 kg/m^2^ (SD = 9.39; range: 25.04–53.40). According to the standard BMI cut-off [52], there were 68 (17.1%) overweight patients and 329 obese patients (82.9%), mostly with severe obesity (49.9%). A total of 126 patients (31.7%) had a BES total score of ≥ 18 (i.e., moderate-to-severe binge eating), and 51 (12.8%) had a BES total score of ≥ 27 (i.e., severe binge eating). Detailed descriptive statistics of the study participants are reported in Table 1.

### 3.1. Internal Consistency and Model Fit of the SCL-K-9

The ordinal α (= 0.91) and inter-item mean index of correlation (*r_ii_* = 0.53) values for the SCL-K-9 were satisfactory. The analysis of the items indicated that all the nine items had acceptable statistics (detailed items statistics are reported in Table 2). The results of the CFA supported a one-factor solution of the SCL-K-9, with all nine items loading significantly on the common latent factor (≥ 0.587). Detailed fit statistics and item loadings are reported in Table 3.

### 3.2. Psychometric Properties of the SCL-K-9

The GSI-K-9 shared 90% of its variance with the original GSI-90 (*r* = 0.95; *p* < 0.001). The convergent validity of the SCL-K-9 was satisfactory. The GSI-K-9 was strongly correlated with the BES total score (*r* = 0.53; *p* < 0.001), as well as with all of the SCL-90-R subscales (*r* > 0.68; *p* < 0.001). No significant correlations were reported with either age (*r* = 0.02; *p* = 0.64) or BMI (*r* = 0.06; *p* = 0.23). However, compared to men, women reported higher scores in the both GSI (0.55 ± 0.53 vs 0.71 ± 0.58 vs.; t _395_ = −2.50; *p* = 0.013) and GSI-K-9 (0.62 ± 0.64 vs 0.85 ± 0.77; t _395_ = −2.73; *p* = 0.007).

**Figure 1:**
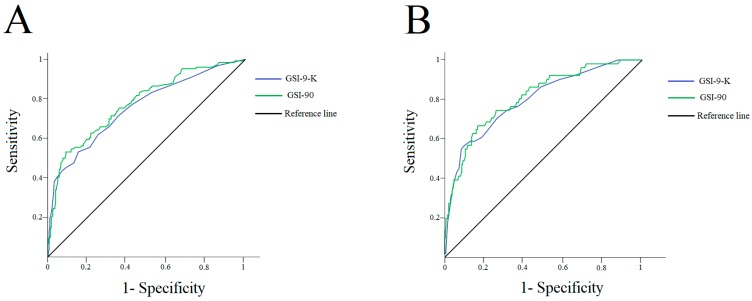
Panel **A**: ROC curve graph for the ability of the GSI-K-9 and GSI-90 to discriminate individuals with moderate-to-severe levels of binge eating (BES total score ≥ 18) from those without a significant level of binge eating symptoms (BES total score < 18). Panel **B**: ROC curve graph for the ability of the GSI-K-9 and GSI-90 to discriminate individuals with severe levels of binge eating (BES total score ≥ 27) from those without significant levels of binge eating symptoms (BES total score < 27). Abbreviations: ROC—receiver operating characteristic; GSI-K-9—global severity index of the Symptom Checklist-K-9; BES—Binge Eating Scale; GSI-90—global severity index of the Symptom Checklist-90-Revised.

A ROC curve procedure showed that both the GSI-K-9 (area under the ROC curve = 0.75, 95%; confidence interval [0.70, 0.81], SE = 0.03, *p* < 0.001) and the GSI-90 (area under the ROC curve = 0.78, 95%; confidence interval [0.73, 0.83], SE = 0.03, *p* < 0.001) classified patients with moderate-to-severe levels of binge eating (i.e., BES total score ≥ 18; Figure 1A). Particularly, a score of 0.83 or higher on the GSI-9-K (Youden index = 0.36) categorized individuals with a sensitivity of 0.62 (62% of all the subjects with BES total score ≥ 18 were correctly detected) and a specificity of 0.75 (25% of patients were incorrectly identified as having a moderate-to-severe level of binge eating). The ROC curve procedure also showed that both the GSI-K-9 (area under the ROC curve = 0.80, 95%; confidence interval [0.73, 0.87], SE = 0.04, *p* < 0.001) and the GSI-90 (area under the ROC curve = 0.81, 95%; confidence interval [0.75, 0.88], SE = 0.03, *p* < 0.001) classified patients with severe levels of binge eating (i.e., BES total score ≥ 27; Figure 1B). Particularly, a score of 0.96 or higher on the GSI-K-9 (Youden index = 0.44) categorized individuals with a sensitivity of 0.71 (71% of all the subjects with BES total score ≥ 27 were correctly detected) and a specificity of 0.73 (27% of patients were incorrectly identified as having a severe level of binge eating).

## 4. Discussion

The major purpose of the current research was to explore the dimensionality and psychometric properties of the SCL-K-9 in a clinical sample of obese and overweight patients seeking weight-loss treatments (i.e., bariatric surgery and nutritional weight-loss therapy). Consistent with previous studies in the general population [30,31], our data supported unidimensionality of the SCL-K-9, with satisfactory psychometric properties. Specifically, the internal consistency, correlations with the original indices of the SCL-90-R, and the convergent validity regarding binge eating severity were all satisfactory. This was in accordance with previous reports detecting the association between binge eating and psychopathology in overweight/obese patients [53,54,55]. Although causal relationships between investigated variables could not be established in our study, it has been proposed that a lack of control over eating could reflect dysfunctional coping strategies consistent with people attempting to self-medicate in response to a range of psychological symptoms and negative affective states [55,56,57]. In line with previous studies [29,31], our results also showed that, compared to men, women exhibited higher scores on the SCL-K-9, suggesting that, among individuals with obesity, psychopathology may be more strongly associated with women than men [10,58,59].

Our data also suggested that the SCL-K-9 could discriminate between patients with and without significant binge eating pathology according to different levels of severity. The ability of the GSI-K-9 to discriminate between individuals with different levels of binge eating was comparable with that of the original GSI-90. For example, a randomly chosen patient with severe binge eating (i.e., BES total score ≥ 27) had an 80% probability of having a higher score on the GSI-K-9 than a randomly chosen individual with a BES total score < 27. This performance was comparable to that observed using the full SCL-90-R (i.e., 81% probability). 

Although the present data are interesting, some limits should be underlined and considered in order to guide future research. One limitation that must be considered is that the high female proportion of our sample limits the generalizability of these findings. Second, we did not investigate the stability of SCL-K-9 over time and its predictive validity on future weight-loss outcomes, such as drop-out rates. Lastly, we used only self-reported measures; in the future, the performance of the SCL-K-9 to classify patients with and without mental disorders should be analyzed using structured clinical interviews. Indeed, although the SCL-90-R is an adequate instrument to assess general psychopathology, it does not diagnose a mental disorder [60].

Despite these limitations, to the best of our knowledge, this is the first study investigating the dimensionality and psychometric properties of the SCL-K-9 in a clinical sample of obese and overweight patients seeking weight-loss treatment.

## 5. Conclusions

Overall, our data suggested that the SCL-K-9 possesses satisfactory psychometric properties and can been used as a short screening tool for the assessment of general psychopathology in overweight/obese patients seeking weight-loss treatment when time restraints preclude the use of the full-length form. As a time-saving instrument, the SCL-K-9 could be useful for clinicians as a first step of a more in-depth assessment procedure, as well as to measure outcomes of psychopathological symptoms associated with weight-loss treatment during follow-up assessment [31]. The ability of this brief version to discriminate between overweight/obese patients with and without significant binge eating pathology may have important clinical implications. Considering that the presence of both pre- and post-operative binge eating negatively influences the long-term outcomes [17,61,62,63], this brief tool could help clinicians to promptly identify high-risk individuals. Accordingly, individuals presenting positive screening results could be addressed via a proper psychosocial intervention [64,65], and surgeons may select a more suitable type of intervention based on the patient’s eating disorder [66]. Finally, the SCL-K-9 could be useful for researchers as a brief psychopathological screening questionnaire in large epidemiological cohort studies.

## Figures and Tables

**Table 1:** Descriptive statistics of the sample.

Variables	Nonsurgical Patients (*N* = 122)	Surgical Candidates (*N* = 275)	Total (*N* = 397)
Age—M ± SD	41.92 ± 13.53	44.08 ± 11.23	43.40 ± 12.02
Females—N (%)	90 (73.8)	201 (73.1)	291 (73.3)
BMI—M ± SD	31.72 ± 6.59	44.15 ± 7.79	40.33 ± 9.39
BMI ≥ 25-29.9 kg/m^2^—N (%)	68 (55.7)	-	68 (17.1)
BMI ≥ 30-34.9 kg/m^2^—N (%)	18 (14.8)	16 (5.8)	34 (8.6)
BMI ≥ 35-39.9 kg/m^2^—N (%)	22 (18.0)	75 (27.3)	97 (24.4)
BMI ≥ 40 kg/m^2^—N (%)	14 (11.5)	184 (66.9)	198 (49.9)
BES—M ± SD	13.50 ± 9.30	14.07 ± 10.28	13.89 ± 9.39
BES total score ≤ 17—N (%)	83 (68)	188 (68.4)	271 (68.3)
BES total score ≥ 18—N (%)	39 (32)	87 (31.6)	126 (31.7)
BES total score ≥ 27—N (%)	14 (11.5)	37 (13.5)	51 (12.8)
GSI-90—M ± SD	0.72 ± 0.59	0.65 ± 0.55	0.67 ± 0.57
Somatization—M ± SD	0.83 ± 0.70	0.97 ± 0.69	0.93 ± 0.70
Obsessive–compulsive symptoms—M ± SD	0.89 ± 0.75	0.66 ± 0.66	0.73 ± 0.69
Interpersonal sensitivity—M ± SD	0.76 ± 0.78	0.68 ± 0.70	0.70 ± 0.73
Depression—M ± SD	0.87 ± 0.78	0.79 ± 0.74	0.81 ± 0.76
Anxiety—M ± SD	0.70 ± 0.65	0.56 ± 0.59	0.60 ± 0.61
Hostility—M ± SD	0.65 ± 0.70	0.44 ± 0.55	0.50 ± 0.61
Phobic anxiety—M ± SD	0.28 ± 0.52	0.31 ± 0.51	0.30 ± 0.52
Paranoid ideation—M ± SD	0.74 ± 0.69	0.59 ± 0.61	0.64 ± 0.64
Psychoticism—M ± SD	0.45 ± 0.56	0.34 ± 0.49	0.37 ± 0.52
GSI-9-K—M ± SD	0.87 ± 0.82	0.74 ± 0.70	0.79 ± 0.74

Abbreviations: BMI—body mass index; GSI-90—global severity index of the Symptom Checklist-90-Revised; GSI-9-K—global severity index of the Symptom Checklist-K-9; BES—Binge Eating Scale.

**Table 2:** Items statistics of the SCL-K-9.

Item Number	Mean	SD	Skewness	Kurtosis	Selectivity	Ordinal Alpha if Item is Dropped	Not at All (%)	A Little Bit (%)	Moderately (%)	Quite a Bit (%)	Extremely (%)
#1	0.53	0.95	1.96	3.26	0.69	0.90	69.3	17.1	7.3	4.3	2.0
#2	0.85	1.09	1.23	0.77	0.71	0.90	51.5	24.1	15.6	5.0	3.8
#3	1.07	1.20	0.97	−0.06	0.72	0.90	42.2	29.4	13.1	10.1	5.3
#4	0.82	1.08	1.28	0.83	0.77	0.90	52.8	25.6	11.6	7.0	3.0
#5	0.61	0.99	1.72	2.31	0.72	0.90	64.3	19.8	8.8	4.8	2.3
#6	0.91	1.05	1.03	0.32	0.76	0.90	46.0	28.9	15.8	7.0	2.3
#7	1.34	1.32	0.68	−0.75	0.55	0.91	34.7	28.9	13.6	13.6	9.3
#8	0.27	0.67	2.82	8.04	0.62	0.91	82.4	11.1	4.0	2.3	0.3
#9	0.66	0.97	1.52	1.69	0.72	0.90	59.5	23.4	10.3	5.0	1.8
Total	0.78	0.74	-	-	-	-					

**Table 3:** Model fit of the SCL-K-9.

Model Fit	χ^2^	df	Sig.	RMSEA	TLI	WRMR			
	34.87	27	0.14	0.027 (0.00/0.05)	0.996	0.50			
Items	#1	#2	#3	#4	#5	#6	#7	#8	#9
Loadings	0.728	0.743	0.775	0.819	0.764	0.797	0.587	0.683	0.763
R^2^	0.530	0.553	0.601	0.671	0.584	0.635	0.344	0.467	0.582

## References

[1] Bersani F.S., Coviello M., Imperatori C., Francesconi M., Hough C.M., Valeriani G., De Stefano G., Bolzan Mariotti Posocco F., Santacroce R., Minichino A. (2015). Adverse psychiatric effects associated with herbal weight-loss products. Biomed. Res. Int..

[2] Chooi Y.C., Ding C., Magkos F. (2019). The epidemiology of obesity. Metabolism.

[3] Lawrence V.J., Kopelman P.G. (2004). Medical consequences of obesity. Clin. Dermatol..

[4] Bray G.A. (2004). Medical consequences of obesity. J. Clin. Endocrinol. Metab..

[5] McCuen-Wurst C., Ruggieri M., Allison K.C. (2018). Disordered eating and obesity: Associations between binge-eating disorder, night-eating syndrome, and weight-related comorbidities. Ann. N. Y. Acad. Sci..

[6] Dawes A.J., Maggard-Gibbons M., Maher A.R., Booth M.J., Miake-Lye I., Beroes J.M., Shekelle P.G. (2016). Mental health conditions among patients seeking and undergoing bariatric surgery: A meta-analysis. JAMA.

[7] Vamado P.J., Williamson D.A., Bentz B.G., Ryan D.H., Rhodes S.K., O’Neil P.M., Sebastian S.B., Barker S.E. (1997). Prevalence of binge eating disorder in obese adults seeking weight loss treatment. Eat. Weight Disord..

[8] Imperatori C., Innamorati M., Lamis D.A., Contardi A., Continisio M., Castelnuovo G., Manzoni G.M., Fabbricatore M. (2016). Factor structure of the binge eating scale in a large sample of obese and overweight patients attending low energy diet therapy. Eur. Eat. Disord. Rev..

[9] Lin H.Y., Huang C.K., Tai C.M., Kao Y.H., Tsai C.C., Hsuan C.F., Lee S.L., Chi S.C., Yen Y.C. (2013). Psychiatric disorders of patients seeking obesity treatment. BMC Psychiatry.

[10] Bianciardi E., Di Lorenzo G., Niolu C., Betro S., Zerbin F., Gentileschi P., Siracusano A. (2019). Body image dissatisfaction in individuals with obesity seeking bariatric surgery: Exploring the burden of new mediating factors. Riv. Psichiatr..

[11] Chan C., Napolitano M.A., Foster G.D., Allison D.B., Baskin M.L. (2009). Assessment of general personality and psychopathology among persons with eating and weight-related concerns. Handbook of Assessment Methods for Eating Behaviors and Weight-Related Problems: Measures, Theory, and Research.

[12] Mechanick J.I., Kushner R.F., Sugerman H.J., Gonzalez-Campoy J.M., Collazo-Clavell M.L., Spitz A.F., Apovian C.M., Livingston E.H., Brolin R., Sarwer D.B. (2009). American association of clinical endocrinologists, the obesity society, and american society for metabolic & bariatric surgery medical guidelines for clinical practice for the perioperative nutritional, metabolic, and nonsurgical support of the bariatric surgery patient. Obesity.

[13] Marek R.J., Ben-Porath Y.S., Heinberg L.J. (2016). Understanding the role of psychopathology in bariatric surgery outcomes. Obes. Rev..

[14] Mauro M., Papelbaum M., Brasil M.A.A., Carneiro J.R.I., Coutinho E.S.F., Coutinho W., Appolinario J.C. (2019). Is weight regain after bariatric surgery associated with psychiatric comorbidity? A systematic review and meta-analysis. Obes. Rev..

[15] De Panfilis C., Cero S., Dall’Aglio E., Salvatore P., Torre M., Maggini C. (2007). Psychopathological predictors of compliance and outcome in weight-loss obesity treatment. Acta Biomed..

[16] Peckmezian T., Hay P. (2017). A systematic review and narrative synthesis of interventions for uncomplicated obesity: Weight loss, well-being and impact on eating disorders. J. Eat. Disord..

[17] Sarwer D.B., Allison K.C., Wadden T.A., Ashare R., Spitzer J.C., McCuen-Wurst C., LaGrotte C., Williams N.N., Edwards M., Tewksbury C. (2019). Psychopathology, disordered eating, and impulsivity as predictors of outcomes of bariatric surgery. Surg. Obes. Relat. Dis..

[18] Wimmelmann C.L., Dela F., Mortensen E.L. (2014). Psychological predictors of weight loss after bariatric surgery: A review of the recent research. Obes. Res. Clin. Pract..

[19] Sheets C.S., Peat C.M., Berg K.C., White E.K., Bocchieri-Ricciardi L., Chen E.Y., Mitchell J.E. (2015). Post-operative psychosocial predictors of outcome in bariatric surgery. Obes. Surg..

[20] Colombo O., Ferretti V.V., Ferraris C., Trentani C., Vinai P., Villani S., Tagliabue A. (2014). Is drop-out from obesity treatment a predictable and preventable event?. Nutr. J..

[21] Altamura M., Porcelli P., Fairfield B., Malerba S., Carnevale R., Balzotti A., Rossi G., Vendemiale G., Bellomo A. (2018). Alexithymia predicts attrition and outcome in weight-loss obesity treatment. Front. Psychol..

[22] Devlin M.J., King W.C., Kalarchian M.A., Hinerman A., Marcus M.D., Yanovski S.Z., Mitchell J.E. (2018). Eating pathology and associations with long-term changes in weight and quality of life in the longitudinal assessment of bariatric surgery study. Int. J. Eat. Disord..

[23] Conceicao E.M., Utzinger L.M., Pisetsky E.M. (2015). Eating disorders and problematic eating behaviours before and after bariatric surgery: Characterization, assessment and association with treatment outcomes. Eur. Eat. Disord. Rev..

[24] Derogatis L. (1977). The scl-90-r Manual.

[25] Ransom D., Ashton K., Windover A., Heinberg L. (2010). Internal consistency and validity assessment of scl-90-r for bariatric surgery candidates. Surg. Obes. Relat. Dis..

[26] Dalle Grave R., Calugi S., Petroni M.L., Di Domizio S., Marchesini G. (2010). Weight management, psychological distress and binge eating in obesity. A reappraisal of the problem. Appetite.

[27] Sarno I., Preti E., Prunas A., Madeddu F. (2011). Scl-90-r: Symptom Checklist 90 r. Versione Italiana Validata e Standardizzata.

[28] Prinz U., Nutzinger D.O., Schulz H., Petermann F., Braukhaus C., Andreas S. (2013). Comparative psychometric analyses of the scl-90-r and its short versions in patients with affective disorders. BMC Psychiatry.

[29] Klaghofer R., Brähler E. (2001). Konstruktion und teststatistische prüfung einer kurzform der scl-90–r [construction and test statistical evaluation of a short version of the scl-90–r]. Z. Klin. Psychol. Psychiatr. Psychother..

[30] Sereda Y., Dembitskyi S. (2016). Validity assessment of the symptom checklist scl-90-r and shortened versions for the general population in ukraine. BMC Psychiatry.

[31] Petrowski K., Schmalbach B., Kliem S., Hinz A., Brahler E. (2019). Symptom-checklist-k-9: Norm values and factorial structure in a representative german sample. PLoS ONE.

[32] Dalle Grave R., Cuzzolaro M., Calugi S., Tomasi F., Temperilli F., Marchesini G. (2007). The effect of obesity management on body image in patients seeking treatment at medical centers. Obesity.

[33] Gormally J., Black S., Daston S., Rardin D. (1982). The assessment of binge eating severity among obese persons. Addict. Behav..

[34] Bianciardi E., Fabbricatore M., Di Lorenzo G., Innamorati M., Tomassini L., Gentileschi P., Niolu C., Siracusano A., Imperatori C. (2019). Prevalence of food addiction and binge eating in an italian sample of bariatric surgery candidates and overweight/obese patients seeking low-energy-diet therapy. Riv. Psichiatr..

[35] Prunas A., Sarno I., Preti E., Madeddu F., Perugini M. (2012). Psychometric properties of the italian version of the scl-90-r: A study on a large community sample. Eur. Psychiatry.

[36] Marcus M.D., Wing R.R., Hopkins J. (1988). Obese binge eaters: Affect, cognitions, and response to behavioural weight control. J. Consult. Clin. Psychol..

[37] Grupski A.E., Hood M.M., Hall B.J., Azarbad L., Fitzpatrick S.L., Corsica J.A. (2013). Examining the binge eating scale in screening for binge eating disorder in bariatric surgery candidates. Obes. Surg..

[38] Marek R.J., Tarescavage A.M., Ben-Porath B.S., Ashton K., Heinberg L.J. (2015). Replication and evaluation of a proposed two-factor binge eating scale (bes) structure in a sample of bariatric surgery candidates. Surg. Obes. Relat. Dis..

[39] Hood M.M., Grupski A.E., Hall B.J., Ivan I., Corsica J. (2013). Factor structure and predictive utility of the binge eating scale in bariatric surgery candidates. Surg. Obes. Relat. Dis..

[40] Timmerman G.M. (1999). Binge eating scale: Further assesment of validity and reliability. J. Appl. Biobehav. Res..

[41] Zumbo B.D., Gadermann A.M., Zeisser C. (2007). Ordinal versions of coefficients alpha and theta for likert rating scales. J. Mod. Appl. Stat. Methods.

[42] Muthén L.K., Muthén B.O. (1998). Mplus User’s Guide.

[43] Mardia K.V. (1970). Measures of multivariate skewnees and kurtosis with applications. Biometrika.

[44] Browne M.W., Cudek R., Long J.S. (1993). Alternative ways of assessing model fit. Testing Structural Equation Models.

[45] Yu C.Y. (2002). Evaluating Cutoff Criteria of Model Fit Indices for Latent Variable Models with Binary and Continuous Outcomes.

[46] Hu L.T., Bentler P.M. (1999). Cutoff criteria for fit indexes in covariance structure analysis: Conventional criteria versus new alternatives. Struct. Equ. Modeling.

[47] Ruopp M.D., Perkins N.J., Whitcomb B.W., Schisterman E.F. (2008). Youden index and optimal cut-point estimated from observations affected by a lower limit of detection. BIOM J..

[48] Fawcett T. (2006). An introduction to roc analysis. Pattern Recogn. Lett..

[49] Centor R.M., Schwartz J.S. (1985). An evaluation of methods for estimating the area under the receiver operating characteristic (roc) curve. Med. Decis. Mak..

[50] Swets J.A. (1988). Measuring the accuracy of diagnostic systems. Science.

[51] Youden W.J. (1950). Index for rating diagnostic tests. Cancer.

[52] The World Health Organisation (WHO) Bmi Classifcation. http://apps.who.int/bmi/index.jsp?introPage=intro_3.htm.

[53] Fandino J., Moreira R.O., Preissler C., Gaya C.W., Papelbaum M., Coutinho W.F., Appolinario J.C. (2010). Impact of binge eating disorder in the psychopathological profile of obese women. Compr. Psychiatry.

[54] Didie E.R., Fitzgibbon M. (2005). Binge eating and psychological distress: Is the degree of obesity a factor?. Eat. Behav..

[55] Imperatori C., Innamorati M., Contardi A., Continisio M., Tamburello S., Lamis D.A., Tamburello A., Fabbricatore M. (2014). The association among food addiction, binge eating severity and psychopathology in obese and overweight patients attending low-energy-diet therapy. Compr. Psychiatry.

[56] Imperatori C., Fabbricatore M., Vumbaca V., Innamorati M., Contardi A., Farina B. (2016). Food addiction: Definition, measurement and prevalence in healthy subjects and in patients with eating disorders. Riv. Psichiatr..

[57] Heatherton T.F., Baumeister R.F. (1991). Binge eating as escape from self-awareness. Psychol. Bull..

[58] Desai R.A., Manley M., Desai M.M., Potenza M.N. (2009). Gender differences in the association between body mass index and psychopathology. CNS Spectr..

[59] Leung S.E., Wnuk S., Jackson T., Cassin S.E., Hawa R., Sockalingam S. (2019). Prospective study of attachment as a predictor of binge eating, emotional eating and weight loss two years after bariatric surgery. Nutrients.

[60] Imperatori C., Della Marca G., Brunetti R., Carbone G.A., Massullo C., Valenti E.M., Amoroso N., Maestoso G., Contardi A., Farina B. (2016). Default mode network alterations in alexithymia: An eeg power spectra and connectivity study. Sci. Rep..

[61] Chao A.M., Wadden T.A., Faulconbridge L.F., Sarwer D.B., Webb V.L., Shaw J.A., Thomas J.G., Hopkins C.M., Bakizada Z.M., Alamuddin N. (2016). Binge-eating disorder and the outcome of bariatric surgery in a prospective, observational study: Two-year results. Obesity.

[62] Freire C.C., Zanella M.T., Segal A., Arasaki C.H., Matos M.I.R., Carneiro G. (2020). Associations between binge eating, depressive symptoms and anxiety and weight regain after roux-en-y gastric bypass surgery. Eat. Weight Disord..

[63] Devlin M.J., King W.C., Kalarchian M.A., White G.E., Marcus M.D., Garcia L., Yanovski S.Z., Mitchell J.E. (2016). Eating pathology and experience and weight loss in a prospective study of bariatric surgery patients: 3-year follow-up. Int. J. Eat. Disord..

[64] David L.A., Sijercic I., Cassin S.E. (2020). Preoperative and post-operative psychosocial interventions for bariatric surgery patients: A systematic review. Obes. Rev..

[65] Conceicao E.M., Goldschmidt A. (2019). Disordered eating after bariatric surgery: Clinical aspects, impact on outcomes, and intervention strategies. Curr. Opin. Psychiatry.

[66] Smith K.E., Orcutt M., Steffen K.J., Crosby R.D., Cao L., Garcia L., Mitchell J.E. (2019). Loss of control eating and binge eating in the 7 years following bariatric surgery. Obes. Surg..

